# Carboxylic Acid Transporters in *Candida* Pathogenesis

**DOI:** 10.1128/mBio.00156-20

**Published:** 2020-05-12

**Authors:** Rosana Alves, Maria Sousa-Silva, Daniel Vieira, Pedro Soares, Yasmin Chebaro, Michael C. Lorenz, Margarida Casal, Isabel Soares-Silva, Sandra Paiva

**Affiliations:** a Centre of Molecular and Environmental Biology (CBMA), University of Minho, Campus de Gualtar, Braga, Portugal; b Institute of Science and Innovation for Bio-Sustainability (IB-S), University of Minho, Campus de Gualtar, Braga, Portugal; c Department of Microbiology and Molecular Genetics, McGovern Medical School, University of Texas, Houston, Texas, USA; Universidade de Sao Paulo

**Keywords:** *Candida* species, acetate, candidiasis, carboxylate transporters, lactate

## Abstract

Opportunistic pathogens such as *Candida* species can use carboxylic acids, like acetate and lactate, to survive and successfully thrive in different environmental niches. These nonfermentable substrates are frequently the major carbon sources present in certain human body sites, and their efficient uptake by regulated plasma membrane transporters plays a critical role in such nutrient-limited conditions. Here, we cover the physiology and regulation of these proteins and their potential role in *Candida* virulence. This review also presents an evolutionary analysis of orthologues of the Saccharomyces cerevisiae Jen1 lactate and Ady2 acetate transporters, including a phylogenetic analysis of 101 putative carboxylate transporters in twelve medically relevant *Candida* species. These proteins are assigned to distinct clades according to their amino acid sequence homology and represent the major carboxylic acid uptake systems in yeast. While Jen transporters belong to the sialate:H^+^ symporter (SHS) family, the Ady2 homologue members are assigned to the acetate uptake transporter (AceTr) family. Here, we reclassify the later members as ATO (acetate transporter ortholog). The new nomenclature will facilitate the study of these transporters, as well as the analysis of their relevance for *Candida* pathogenesis.

## Abstract (precis)

Opportunistic pathogens such as *Candida* species can use carboxylic acids, like acetate and lactate, to survive and successfully thrive in different environmental niches. These nonfermentable substrates are frequently the major carbon sources present in certain human body sites, and their efficient uptake by regulated plasma membrane transporters plays a critical role in such nutrient-limited conditions. Here, we cover the physiology and regulation of these proteins and their potential role in *Candida* virulence.

## INTRODUCTION

Transport of carboxylic acids across cell membranes is of critical importance for all living systems, from bacteria to fungi to mammalian cells (1). At physiological pH, these organic compounds prevail in their anionic form, requiring a transport system to cross the plasma membrane; whereas, at lower pH, the undissociated form of the acid can enter the cell by simple diffusion (1, 2). Integral membrane transporters and channels, the two major classes of transport proteins, are thereby important mediators between the intracellular and extracellular environment by precisely controlling the influx of important nutrients or the efflux of unwanted metabolites (3). In Saccharomyces cerevisiae, Jen1 and Ady2 are the main transport systems responsible for the uptake of monocarboxylates at conditions where these substrates can be used as main carbon sources. The aquaglyceroporin channel Fps1 has also been reported to mediate the import of undissociated acetic acid by facilitated diffusion (4), though this seems to depend on the extracellular environment (5). Both Jen1 and Ady2 transporters have also been suggested to be involved in the export of monocarboxylates (6, 7); yet, it is unclear how these efflux processes are regulated.

Opportunistic pathogens can acquire the available carboxylic acids present in the human host, either produced by host microbiota or host cells (8). The uptake of carboxylates, such as lactate and acetate, sustains cell metabolism and growth when other preferable carbon sources are limited. This is particularly relevant for a group of pathogenic *Candida* species, which are well-adapted to different human environmental niches and import these carboxylic acids in order to survive under host conditions that otherwise would be deleterious. These nutrient-restricted environments are predominantly found within the gut and vagina, with the host microbiota having a predominant role in the production of propionate, acetate, and lactate (9, 10). The induction of lactate and acetate transporters in *Candida* upon macrophage or neutrophil internalization also supports the idea that these nutrients are readily available inside phagocytic cells (11–16).

**FIG 1:**
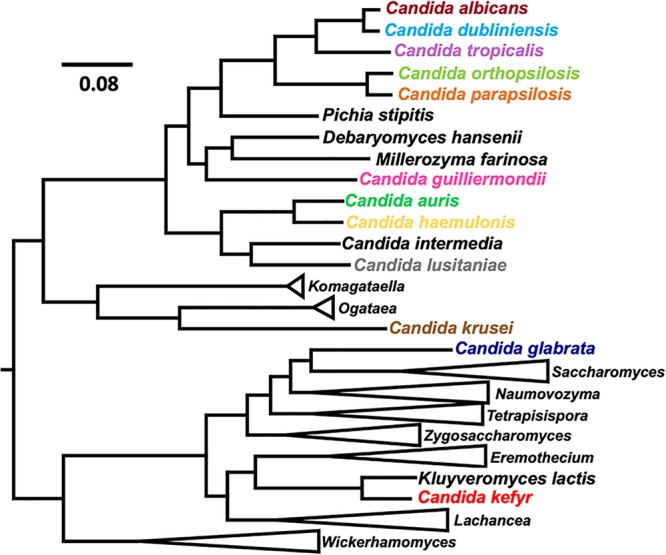
Evolutionary relationship between *Candida* species and other yeasts. Phylogenetic reconstruction was performed using maximum likelihood in IQ-TREE (73), the JTT (Jones-Taylor-Thornton) model of amino acid evolution, and four gamma-distributed rates. Phylogeny was based on a total of 1,567 concatenated proteins within the proteome of the different species. BLAST searches were performed comparing the proteome (obtained in NCBI) of the selected species in order to detect those conserved proteins across a total of 77 fungal species. These proteins are essential proteins beyond the specific biology of the different yeasts offering a clear high-resolution evolutionary view between the different species. All obtained bootstrap values showed 100% in confidence. *Candida species* relevant for this study are highlighted with different colors. Branches that were not relevant were collapsed with the representative genus indicated.

The different species comprising the genus *Candida* are spread among distinct phylogenetic clades, which also include other nonpathogenic species (Fig. 1). This suggests that the ability to infect humans has evolved several times, independently, among this diverse group of yeasts (17). In this review, we revisit the two major families of carboxylic acid transporters in yeast and explore their contribution for *Candida* pathogenesis. The expansion and functional specialization of some members of these families in pathogenic species, such as in Candida albicans and Candida parapsilosis, may have emerged as means of either environmental or host adaptation, increasing the ability of these species to thrive within the human host and consequently account for their increased virulence. To give a better overview on the representation of these transporters in *Candida*, we conducted protein BLAST (BLASTp) searches in NCBI (https://blast.ncbi.nlm.nih.gov) and identified all of the orthologues of the functionally validated S. cerevisiae monocarboxylate transporters (Jen1 and Ady2) for a set of medically relevant *Candida* species. In particular, these two transporters were independently used as BLAST queries in searching the proteome of C. albicans, Candida glabrata, Candida tropicalis, C. parapsilosis, Candida orthopsilosis, Candida dubliniensis, Candida krusei, Candida auris, Candida lusitaniae, Candida haemulonis, Candida guilliermondii, and Candida kefyr. This set includes the most studied and well-characterized *Candida* species but also infrequent species that are emerging as successful pathogens. Finally, we reclassified and renamed some of these homologues based on recently reported structural and functional data. In particular, we propose the exclusive use of ATO (acetate transporter ortholog) for all of the yeast Ady2 ortholog members of the acetate uptake transporter (AceTr) family to better describe their function as acetate transporters (18–20).

## PHYLOGENETIC EVOLUTION OF JEN TRANSPORTERS

The lactate transporter Jen1 was the first monocarboxylate transporter identified in fungi (21). This proton-coupled monocarboxylate transporter is a member of the major facilitator superfamily (MFS) (TCDB 2.A.1.12.2) (22), which is responsible for the active transport of lactate, pyruvate, acetate, propionate (21, 23, 24), and also selenite (25). So far, among all *Candida* species, only two *S. cerevisiae* Jen1 (ScJen1) orthologues were functionally characterized in C. albicans, one encoding a monocarboxylate transporter (CaJen1) for lactate, pyruvate, and propionate (26) and the other encoding a dicarboxylate transporter (CaJen2) for succinate and malate (13). Both CaJen1 and CaJen2 transporters are tightly regulated by different carbon sources, being repressed by glucose and induced by their specific substrates (13). This upregulation was also confirmed *in vivo*, in C. albicans cells infecting the kidney, in a murine model of systemic candidiasis, and upon phagocytosis by murine macrophages or human neutrophils (13).

**FIG 2:**
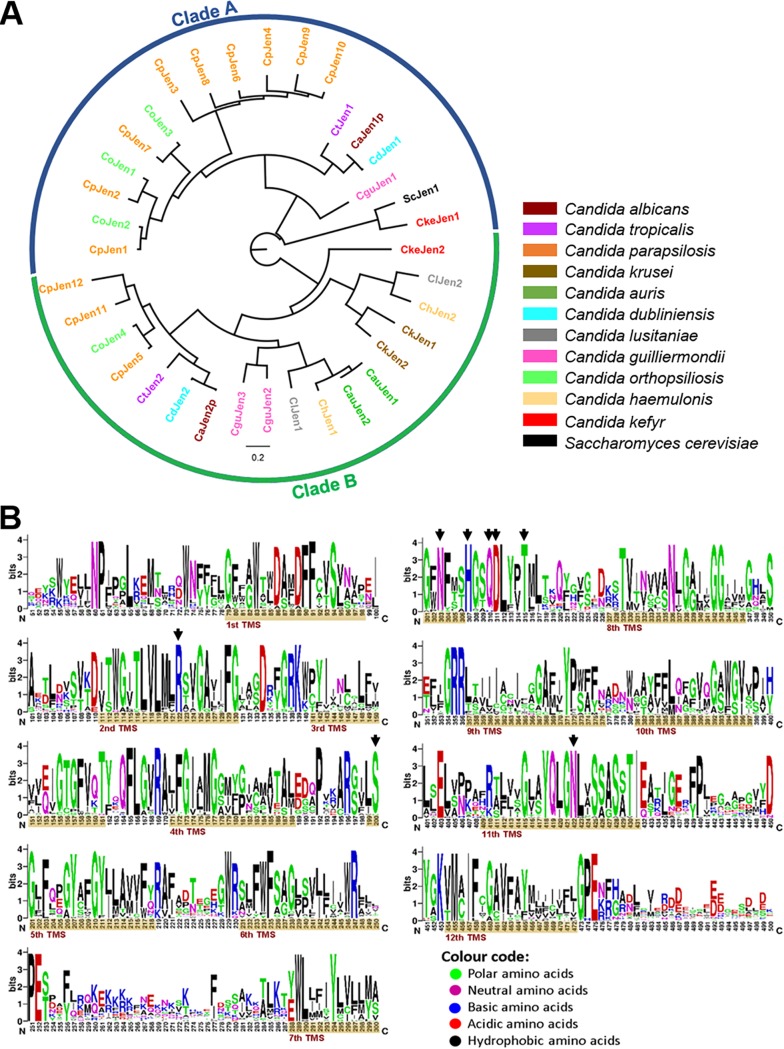
Evolutionary analysis of S. cerevisiae Jen homologues in *Candida* species. (A) A multiple alignment with the 36 ScJen1 homologues found in *Candida* species was performed using MAFFT v.7 (74) and checked manually for incongruences. The alignments were then used in the phylogenetic reconstruction. A maximum-likelihood approach, suitable for deep evolution, was conducted in MEGA7 (75) using the JTT model of amino acid evolution, four gamma-distributed rates, and a site coverage cutoff of 90%. A total of 250 replications were performed for the bootstrap analysis. Names of the homologues are colored according to the species where they were detected. Output trees were edited in FigTree v.1.4.4. (http://tree.bio.ed.ac.uk/software/figtree/). (B) Conservation logo of the alignment that displayed residues in 90% of the sequences using WebLogo (https://weblogo.berkeley.edu). Transmembrane segments (TMSs) predicted with TMHMM 2.0 (http://www.cbs.dtu.dk/services/TMHMM/) are highlighted as brown bars in the logo, and black arrows indicate amino acids belonging to previously identified functional domains (32, 33).

The phylogenetic analysis of ScJen1 was performed with the available amino acid sequences for several *Candida* species. The obtained tree displays 36 Jen-like proteins and suggests the existence of two functional clusters, which we designated as clade A and clade B (Fig. 2A; see also Table S1 in the supplemental material). While clade A comprises the functionally characterized CaJen1 monocarboxylate transporter, clade B contains the CaJen2 dicarboxylate transporter (Fig. 2A). The branching pattern within the two clades is consistent with the species tree topology (see Fig. 1 for reference tree). With the exception of C. glabrata, which does not have any Jen transporters, all *Candida* species analyzed have at least two ScJen1 orthologues. The absence of Jen transporters has already been reported for other *Saccharomycetaceae* yeasts that diverged after the whole-genome duplication (27). Curiously, though, lactate assimilation is required for C. glabrata survival in the intestine, as mutants in the lactate dehydrogenase Cyb2 are rapidly outcompeted (28). C. parapsilosis, C. orthopsilosis, and C. guilliermondii contain, respectively, twelve, four, and three ScJen1 homologues, which are not equally distributed within the two clades (Fig. 2A). In the case of C. parapsilosis and C. orthopsilosis, this observation is consistent with the high level of genomic variation displayed by these species, including copy number variations, a phenomenon likely due to selective pressures present in the environment rather than in the human host (29). This phenomenon was also reported for Yarrowia lipolytica, which has 6 ScJen1 homologues. A phylogenetic analysis predicted that 12 duplications and losses occurred during evolution in the *Yarrowia* Jen clade (27). Moreover, the ScJen1 orthologues found in C. krusei, C. auris, C. lusitaniae, and C. haemulonis are exclusively clustered in clade B (Fig. 2A). While these clades were initially assumed to be two different functional clusters in fungi, with the Jen1 cluster comprising only monocarboxylate transporter proteins and the Jen2 containing dicarboxylate transporters (1, 30), recent evidence has shown that members of both clades may have overlapping substrate specificities (31). The possibility that an ancestral Jen transporter encoded a promiscuous protein with the ability of transporting both mono- and dicarboxylates is highly plausible, but it still remains unclear (30). Studies that have attempted to reconstruct the evolutionary origin of the Jen family suggest *JEN2* as the ancestral gene (27, 30). However, due to a dynamic evolutionary history of subsequent duplications and losses, other members of this family, such as *JEN1*, have emerged (27, 30).

10.1128/mBio.00156-20.1 TABLE S1 GenBank accession numbers of ScJen1 homologues present in eleven *Candida* species with annotated/former designations and the following suggested designation based on ScJen1 gene homology. Download Table S1, DOCX file, 0.02 MB. Copyright © 2020 Alves et al. 2020 Alves et al. This content is distributed under the terms of the Creative Commons Attribution 4.0 International license.

The predicted three-dimensional structure of ScJen1, consisting of 12 transmembrane segments (TMSs), resembles the common topology of other MFS members. Structure-function studies, benefiting from the solved structure of other MFS members, have identified several conserved amino acids that are essential for the binding and cotranslocation of the substrates (32, 33). When analyzing the amino acid sequences, it is possible to observe that most of the functional motifs and amino acids described for ScJen1 are highly conserved across all of the identified orthologues (Fig. 2B). This observation suggests that these transporters are likely to retain the same function. For instance, the conserved motif NXX(S/T)HX(S/T)QDXXXT, located at the end of the putative seventh TMS, has been reported as part of the translocation pathway (33). Protein modeling has demonstrated that the conserved amino acids of this motif face an internal pore and potentially interact with the carboxylic substrates, which is consistent with their crucial roles in protein activity, transport ability and specificity, as well as substrate affinity (32, 33). Other polar conserved residues located at different TMSs have also been identified to be part of the pore and play special roles in substrate specificity by allowing the discrimination between mono- and dicarboxylates (32).

## PHYLOGENETIC EVOLUTION OF ATO TRANSPORTERS

The first member of the acetate transporter family to be reported in the literature was the yeast Yarrowia lipolytica Gpr1 protein (34, 35), which was later confirmed to be an acetate transporter (18). Since then, many orthologues have been identified in the three domains of life (18). Some examples include S. cerevisiae Ady2 (ScAdy2) and Aspergillus nidulans AcpA in eukarya (36, 37), AceP in archaea (18, 38), and SatP in bacteria (39). Although well-represented among the different taxonomic groups, these orthologues seem almost ubiquitous in fungi (18). This observation suggests that acetate transporters play key roles in fungal species, which is in agreement with their involvement in essential physiological processes, such as ascus formation in S. cerevisiae or germination of conidiophores in Aspergillus nidulans (40). In S. cerevisiae, Ady2 has been identified as an acetate permease with the ability to transport other monocarboxylates, such as propionate, formate, and lactate (18, 36). Besides *ADY2* (also known as *ATO1*), the genome of S. cerevisiae harbors two additional homologues, *FUN34* (also known as *ATO2*) and *ATO3*. These three transporters are highly induced under carbon-limiting conditions and during the stationary phase of glucose grown cells (41, 42). The deletion of Sc*ADY2* results in the loss of mediated acetate uptake at pH 6.0 (36).

ScAdy2 (named originally for “accumulation of dyads 2”) was also reported to be expressed during meiosis, which is required for the regulation of meiotic plaque components in sporulation (43). Mutants in Sc*ADY2* predominantly form dyads and display a decreased spore formation (44). The standard formulation for inducing sporulation in yeast contains acetate as the primary carbon source, which probably links the molecular function of the Ady2 protein to this phenotype (44, 45).

These transporters were reported to be involved in ammonium export, hence the acronym ATO, which stands for “ammonia transport outward,” (39) was adopted. In S. cerevisiae, this volatile alkaline compound is transmitted between yeast colonies as a signal to inhibit growth of the facing parts of both colonies, a signaling process that requires amino acid uptake (46). The three Ato transporters were suggested to act as ammonium/H^+^ antiporters by extruding ammonium and importing protons and, thus, contributing to the increase of external pH observed during ammonia signaling (47). The association of the Ato proteins with ammonium transport is genetic, and there is no homology with bona fide ammonium transporters nor any biochemical evidence that they transport ammonia or ammonium.

These transporters were recently reclassified into the acetate uptake transporter (AceTr) family (TCDB 2.A.96) (22) based on the characterization of the abovementioned orthologues, from all domains of life, as acetate transporters (18–20, 37, 39). The confusing nomenclature, often deriving from phenotypes that are indirectly linked to function (ADY, FUN, ATO), represents a barrier to understanding and studying this interesting family. We, therefore, propose redefining the Ady2/Ato proteins as “acetate transport ortholog” instead of the previous designation as “ammonia transport outward” to better reflect and describe their function as acetate transporters, while still maintaining some consistency with the literature (see Table S2 in the supplemental material).

10.1128/mBio.00156-20.2 TABLE S2 GenBank accession numbers of ScAdy2 homologues present in twelve *Candida* species with annotated/former designations and the following suggested designation based on ScAdy2 gene homology. Download Table S2, DOCX file, 0.03 MB. Copyright © 2020 Alves et al. 2020 Alves et al. This content is distributed under the terms of the Creative Commons Attribution 4.0 International license.

**FIG 3:**
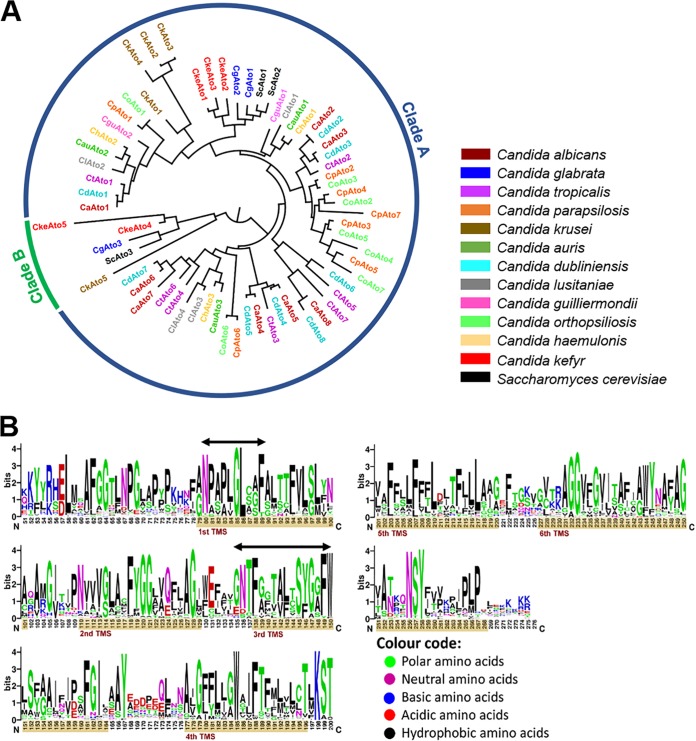
Evolutionary analysis of S. cerevisiae Ato homologues in *Candida* species. (A) A multiple alignment with the 65 ScAto1 homologues found in *Candida* species was performed using MAFFT v.7 (74) and checked manually for incongruences. The alignments were then used in the phylogenetic reconstruction. A maximum-likelihood approach, suitable for deep evolution, was conducted in MEGA7 (75) using the JTT model of amino acid evolution, four gamma-distributed rates, and a site coverage cutoff of 90%. A total of 250 replications were performed for the bootstrap analysis. Names of the homologues are colored according to the species where they were detected. Output trees were edited in FigTree v.1.4.4. (http://tree.bio.ed.ac.uk/software/figtree/). (B) Conservation logo of the alignment that displayed residues in 90% of the sequences using WebLogo (https://weblogo.berkeley.edu). Transmembrane segments (TMSs) predicted with TMHMM 2.0 (http://www.cbs.dtu.dk/services/TMHMM/) are highlighted as brown bars in the logo and arrows indicate amino acids belonging to previously identified functional domains (19).

The phylogenetic analysis of ScAdy2/Ato orthologues (hereafter referred as ScAto1) in the selected *Candida* species resulted in a phylogenetic tree containing 65 Ato-like proteins organized into two asymmetrical clusters (Fig. 3A). Cluster A includes most of the *Candida* orthologues, while cluster B contains only four orthologues and one homologue of ScAto1. All of the selected *Candida* species have at least two orthologues, and more than 50% of the species have five or more orthologues (Fig. 3A), which is in agreement with the high frequency of these proteins in fungal organisms (18). In particular, the Ato family in C. albicans is greatly expanded when compared with other species, being composed of 10 Ato-like proteins (48). However, two of these putative acetate transporters (CaAto9 and CaAto10) (see Table S2) were shorter than the other retrieved sequences. The examination of the BLAST results and the genomic position of each homologue suggests that these were originally the same gene split by a transposable element. The independent alignment of CaAto9 and CaAto10 with the remaining homologues revealed that they align consecutively to each other. Furthermore, phylogenetic analyses performed individually on each sequence revealed the same evolutionary position for both CaAto9 and CaAto10 (phylogenetically close to CaAto6 followed by CaAto5). Given this, CaAto9 and CaAto10 were excluded, as they are most probably nonfunctional homologues. Two other sequences, CtAto8 and CguAto3, were relatively shorter than the other sequences in the alignment. Analyses with TMHMM v. 2.0 (http://www.cbs.dtu.dk/services/TMHMM) showed that these proteins only contain 3 and 5 helices, respectively, instead of the expected six detected for this transporter family. The analysis of the genomic sequence following the termination codon of the protein revealed the presence of the homologous absent portion, indicating that the proteins were truncated, likely recently in evolution. As we have no indication if the truncated proteins are fully functional, we excluded these sequences from further analyses.

As mentioned above, hallmarks of this family include six transmembrane domains but also conserved motifs that are involved in the transport mechanism of acetate in Ato members but also succinate in SatPs (18, 19). The Ato1 orthologues in Escherichia coli (ecSatP) and Citrobacter koseri (ckSatP) are so far the only members of the AceTr family whose structures are available (19, 49). A hexameric UreI-like channel structure was reported for ecSatP (49); however, more recently, it was suggested that the closed state of the channel represents a relatively high energy barrier, 15 kcal/mol (20). The signature NPAPLGL(M/S) motif of the AceTr family, located at the beginning of the first TMS (Fig. 3B), has been reported to be essential for substrate uptake (18, 35). Recent structural analyses with the SatP homolog from Citrobacter koseri have demonstrated that these conserved amino acids are in the vicinity of the first and second acetate/succinate-binding sites, allowing the translocation of the substrate (18, 19). The dissection of the molecular mechanism of the acetate transport in several members of the AceTr family suggests that these transporters are energy dependent (18, 20, 36, 39, 50). This functional motif, as well as other amino acids that play important roles in the transport mechanism of Ato transporters, are highly conserved among all of the identified *Candida* orthologues, suggesting that *Candida* Atos are likely to retain the same function (Fig. 3B). However, an extensive phylogenetic analysis has recently assigned all of the known members of this family into two distinct clades—the prokaryotic clade, where SatPs are included, and a eukaryotic clade comprising the Ato transporters (18). This suggests that despite having a conserved motif involved in substrate recognition and transport, the members of these two clades may display different specificities, which is in agreement with what is already described for the Jen1 homologs that present a conserved signature motif and different specificities throughout the distinct family members (1, 31).

## FUNCTIONAL SPECIALIZATION OF JEN AND ATO TRANSPORTERS IN *CANDIDA*

The idea that carboxylates are essential nutrients for the survival of *Candida* species in certain host niches is supported by many studies that have demonstrated that *Candida* cells upregulate several metabolic pathways involved in the utilization of these carbon sources when infecting tissues and organs (12, 51–53). For instance, several studies in *Candida* have consistently demonstrated that both Jen and Ato transporters are strongly upregulated following phagocytosis, reinforcing the idea that carboxylate assimilation is an integral part of the response to phagocytosis (11, 13–16). Considering that inside the phagolysosome glucose levels are low, lactate and acetate, along with amino acids, may be the main sources of acetyl coenzyme A (acetyl-CoA) that feed the glyoxylate cycle, allowing *Candida* survival in this environment (11, 16, 53). Indeed, abrogation of both carboxylic acid and amino acid uptake confers additive defects in virulence in macrophage and mouse models (54). Consistent with this observation, the acetyl-CoA synthetases, which convert acetate into acetyl-CoA, are upregulated after C. albicans phagocytosis by macrophages and neutrophils (12). C. glabrata mutants in Ato1 and Fps1 were reported to be more efficiently internalized and phagocytized by macrophages than wild-type cells in RPMI medium. But the presence of acetic acid rendered the mutant cells more resistant to macrophage killing, showing that this process is dependent on the carbon source. (55). The uptake of carboxylic acids, mediated either by Jen or Ato transporters, results in the alkalization of the extracellular environment in specific growth conditions. The neutralization of the acidic phagolysosome allows C. albicans to readily escape from the immune cells by differentiating into filamentous hyphae and inducing macrophage pyroptosis, a programmed cell death pathway (56–60). Mutations in the C. albicans *ATO1* and *ATO5* impair neutralization, hyphal differentiation, and macrophage killing (48). This is significant, as *Candida* cells after macrophage killing can continue to successfully thrive in the human host.

Noteworthy, the AceTr family is greatly expanded in *Candida*, especially in C. albicans, when compared to other fungi (Fig. 3A). Gene family expansions in these yeasts occur primarily by gene duplication and under stressful environmental conditions and are often associated with pathogenicity (61). Although most of the duplicated genes are often deleted, some may be retained if either gene dosage or functional specialization are advantageous (61–63). Undoubtedly, an increased number of Ato transporters is expected to endow cells with the advantage of being better adapted for human colonization and infection, which correlates well with the pathogenic profile of C. albicans. But why would 8 acetate transporter genes be retained in C. albicans? An analogy could be made with the 20 hexose transporter genes of S. cerevisiae, where different transporters are tuned to different hexose levels presumably under intense competition (64). We would have to assume that the Ato proteins perform important functions for growth or survival. Perhaps some of the Atos have evolved to be sensors like glucose sensors Snf3 and Rgt2 in S. cerevisiae (64). Although both Jen and Ato transporters have been mainly associated with nutrient assimilation, they may also play important roles in cellular homeostasis by mediating the export of lactate (7).

Interestingly, lactate appears to be a signal of the host environment, and its presence, even when glucose is abundant, induces multiple physiological changes relevant to pathogenesis, including resistance to certain stresses and antifungal drugs (65, 66). Some of these changes are mediated via alterations in the cell wall, which then also impacts recognition by phagocytes (67, 68). This is also true in C. glabrata (69). Lactate and amino acids induce distinct patterns of stress tolerance in C. albicans, suggesting that this species has evolved to identify specific nutrients as signals to direct responses to specific host niches (54). These transporters also have been reported to modulate biofilm formation and resistance to antifungal drugs in both C. albicans and C. glabrata (55, 70).

These results taken together suggest that carboxylic acid transporters are important mediators of host-pathogen interactions by allowing rapid adaptation to different environmental conditions and modulating the virulent properties of *Candida* species. Consistent with this idea, dominant mutations have been isolated in the Ato homologues in S. cerevisiae and Y. lipolytica that render cells hypersensitive to acetic acid (71). This sensitivity is seen at acidic pH where acetic acid would freely diffuse into the cell and likely would acidify the cytosol without compensatory responses. This suggests the possibility that the Ato proteins are bidirectional transporters and, in some cases, can pump acetate out of the cell (71). In fact, the only acetate exporter reported in *Candida* species is the C. glabrata drug:H^+^ antiporter (DHA) CgDtr1, which is responsible for the export of acetate and involved in weak acid stress resistance in RPMI medium at pH 4.5 (72). In this study, the tested pH was below the pK_a_ of the acid, conditions where the acid enters the cell mostly by simple diffusion and can impose significant stress in the form of cytosolic acidification. In *Candida* species, no pleiotropic drug resistance (PDR) transporters belonging to the ATP-binding cassette (ABC) superfamily have been so far associated with the export of carboxylates in acid stress conditions. Since weak acid stresses are common in many host niches, the proliferation of the Ato family in pathogenic species may be a response to these stresses.

## CONCLUSIONS AND FUTURE PERSPECTIVES

The ability to transport carboxylates inside the cell represents an advantage for *Candida* when these nutrients are the main exogenous carbon sources available. Jen and Ato family members, in certain conditions, seem to play critical roles in *Candida* pathogenesis, as they allow cells to sustain metabolism and survival when thriving in the human host. However, many details are missing regarding the energetics, specificity, and regulation of these transporters. Further research will be needed to determine individual transport properties, potential redundancies, and functional roles of carboxylate transporters in *Candida* virulence and pathogenesis.
