# Supplementary material for: Carboxylic Acid Transporters in Candida Pathogenesis
Source: mBio. 2020 May 12;11(3):e00156-20. doi: 10.1128/mBio.00156-20 (PMC7218280; doi:10.1128/mBio.00156-20)
Supplement: TABLE S1 [file mBio.00156-20-st001.docx]

**Carboxylic acid transporters in *Candida* pathogenesis**

Rosana Alves^1,2^, Maria Sousa-Silva^1,2^, Daniel Vieira^1,2^, Pedro Soares^1,2^, Yasmin Chebaro^3^, Michael C. Lorenz^3*^, Margarida Casal^1,2^, Isabel Soares-Silva^1,2^ and Sandra Paiva^1,2*^

^1^Centre of Molecular and Environmental Biology (CBMA), University of Minho, Campus de Gualtar, Braga, Portugal.

^2^Institute of Science and Innovation for Bio-Sustainability (IB-S), University of Minho, Campus de Gualtar, Braga, Portugal.

^3^Department of Microbiology and Molecular Genetics, University of Texas, McGovern Medical School, Houston, TX.

***Correspondence:**

Dr. Sandra Paiva, spaiva@bio.uminho.pt

Dr. Michael Lorenz, Michael.Lorenz@uth.tmc.edu

**Table S1. GenBank accession numbers of *Sc*Jen1 homologues present in eleven *Candida* species with annotated/former and the following suggested designation based on *Sc*Jen1 gene homology**

| ***Sc*Jen1 homologs by specie** | **GenBank Ac. no.** | **Former designation \| Alias** | **Suggested annotation** |
| --- | --- | --- | --- |
| *C. albicans* [2] | XP_716108.1 | Jen1p [Candida albicans SC5314] | CaJen1 |
|  | XP_717110.1 | Jen2p [Candida albicans SC5314] | CaJen2 |
| *C. orthopsilosis* [4] | XP_003868359.1 | hypothetical protein CORT_0C00780 [Candida orthopsilosis Co 90-125] | CoJen1 |
|  | XP_003868357.1 | Jen1 protein [Candida orthopsilosis Co 90-125] | CoJen2 |
|  | XP_003868365.1 | tRNA-Arg [Candida orthopsilosis Co 90-125] | CoJen3 |
|  | XP_003869929.1 | Jen2 protein [Candida orthopsilosis Co 90-125] | CoJen4 |
| *C. auris* [2] | PIS50386.1 | hypothetical protein B9J08_004204 [[Candida] auris] | CauJen1 |
|  | PIS50738.1 | uncharacterized protein CJI97_004268 [[Candida] auris] | CauJen2 |
| *C. krusei* [2] | OUT23441.1 | hypothetical protein CAS74_001759 [Pichia kudriavzevii] | CkJen1 |
|  | OUT23260.1 | hypothetical protein CAS74_001578 [Pichia kudriavzevii] | CkJen2 |
| *C. lusitaniae* [2] | XP_002614689.1 | hypothetical protein CLUG_05468 [Clavispora lusitaniae ATCC 42720] | ClJen1 |
|  | XP_002617815.1 | hypothetical protein CLUG_01274 [Clavispora lusitaniae ATCC 42720] | ClJen2 |
| *C. parapsilosis* [12] | CCE42292.1 | hypothetical protein CPAR2_808410 [Candida parapsilosis] | CpJen1 |
|  | CCE42290.1 | hypothetical protein CPAR2_808390 [Candida parapsilosis] | CpJen2 |
|  | CCE44929.1 | hypothetical protein CPAR2_407310 [Candida parapsilosis] | CpJen3 |
|  | CCE44923.1 | hypothetical protein CPAR2_407250 [Candida parapsilosis] | CpJen4 |
|  | CCE44403.1 | hypothetical protein CPAR2_402040 [Candida parapsilosis] | CpJen5 |
|  | CCE44586.1 | hypothetical protein CPAR2_403890 [Candida parapsilosis] | CpJen6 |
|  | CCE42284.1 | hypothetical protein CPAR2_808330 [Candida parapsilosis] | CpJen7 |
|  | CCE44209.1 | hypothetical protein CPAR2_400100 [Candida parapsilosis] | CpJen8 |
|  | CCE44928.1 | hypothetical protein CPAR2_407300 [Candida parapsilosis] | CpJen9 |
|  | CCE44927.1 | hypothetical protein CPAR2_407290 [Candida parapsilosis] | CpJen10 |
|  | CCE44404.1 | hypothetical protein CPAR2_402050 [Candida parapsilosis] | CpJen11 |
|  | CCE40688.1 | hypothetical protein CPAR2_107230 [Candida parapsilosis] | CpJen12 |
| *C. dubliniensis* [2] | XP_002419556.1 | carboxylic acid transporter, putative [Candida dubliniensis CD36] | CdJen1 |
|  | XP_002420040.1 | carboxylic acid transporter protein, putative [Candida dubliniensis CD36] | CdJen2 |
| *C. tropicalis* [2] | XP_002551411.1 | hypothetical protein CTRG_05709 [Candida tropicalis MYA-3404] | CtJen1 |
|  | XP_002545520.1 | hypothetical protein CTRG_00301 [Candida tropicalis MYA-3404] | CtJen2 |
| *C. kefyr* [2] | XP_022677158.1 | uncharacterized protein KLMA_60073 [Kluyveromyces marxianus DMKU3-1042] | CkeJen1 |
|  | XP_022674588.1 | putative sialic acid transporter [Kluyveromyces marxianus DMKU3-1042] | CkeJen2 |
| *C. guilliermondii* [3] | XP_001484059.1 | hypothetical protein PGUG_03440 [Meyerozyma guilliermondii ATCC 6260] | CguJen1 |
|  | XP_001484060.1 | hypothetical protein PGUG_03441 [Meyerozyma guilliermondii ATCC 6260] | CguJen2 |
|  | XP_001486752.1 | hypothetical protein PGUG_00129 [Meyerozyma guilliermondii ATCC 6260] | CguJen3 |
| *C. haemulonis* [2] | XP_025341865.1 | hypothetical protein CXQ85_004438 [[Candida] haemulonis] | ChJen1 |
|  | XP_025341422.1 | hypothetical protein CXQ85_002273 [[Candida] haemulonis] | ChJen2 |
