# Supplementary material for: Carboxylic Acid Transporters in Candida Pathogenesis
Source: mBio. 2020 May 12;11(3):e00156-20. doi: 10.1128/mBio.00156-20 (PMC7218280; doi:10.1128/mBio.00156-20)
Supplement: TABLE S2 [file mBio.00156-20-st002.docx]

**Carboxylic acid transporters in *Candida* pathogenesis**

Rosana Alves^1,2^, Maria Sousa-Silva^1,2^, Daniel Vieira^1,2^, Pedro Soares^1,2^, Yasmin Chebaro^3^, Michael C. Lorenz^3*^, Margarida Casal^1,2^, Isabel Soares-Silva^1,2^ and Sandra Paiva^1,2*^

^1^Centre of Molecular and Environmental Biology (CBMA), University of Minho, Campus de Gualtar, Braga, Portugal.

^2^Institute of Science and Innovation for Bio-Sustainability (IB-S), University of Minho, Campus de Gualtar, Braga, Portugal.

^3^Department of Microbiology and Molecular Genetics, University of Texas, McGovern Medical School, Houston, TX.

***Correspondence:**

Dr. Sandra Paiva, spaiva@bio.uminho.pt

Dr. Michael Lorenz, Michael.Lorenz@uth.tmc.edu

**Table S2. GenBank accession numbers of *Sc*Ady2 homologues present in twelve *Candida* species with annotated/former and the following suggested designation based on *Sc*Ady2 gene homology**

| ***Sc*Ady2 homologs by specie** | **GenBank Ac. no.** | **Former designation** | **Suggested annotation** |
| --- | --- | --- | --- |
| *C. glabrata* [3] | XP_449497.1 | uncharacterized protein CAGL0M03465g [[Candida] glabrata] | CgAto1 |
|  | XP_449115.1 | uncharacterized protein CAGL0L07766g [[Candida] glabrata] | CgAto2 |
|  | XP_444907.1 | uncharacterized protein CAGL0A03212g [[Candida] glabrata] | CgAto3 |
| *C. albicans* [10] | XP_710295.1 | putative ammonium permease [Candida albicans SC5314] | CaAto1 |
|  | XP_710650.1 | Ato1p [Candida albicans SC5314] | CaAto2 |
|  | XP_718515.2 | Ato2p [Candida albicans SC5314] | CaAto3 |
|  | XP_714701.1 | Ato6p [Candida albicans SC5314] | CaAto4 |
|  | XP_714703.1 | Ato5p [Candida albicans SC5314] | CaAto5 |
|  | XP_716748.1 | Frp6p [Candida albicans SC5314] | CaAto6 |
|  | XP_716747.2 | Frp5p [Candida albicans SC5314] | CaAto7 |
|  | XP_019330752.1 | Ato7p [Candida albicans SC5314] | CaAto8 |
|  | XP_717953.1 | Ato10p [Candida albicans SC5314] | CaAto9 |
|  | XP_717951.1 | Ato9p [Candida albicans SC5314] | CaAto10 |
| *C. auris* [3] | XP_028889701.1 | uncharacterized protein CJI97_002436 [[Candida] auris] | CauAto1 |
|  | XP_028890140.1 | uncharacterized protein CJI97_002886 [[Candida] auris] | CauAto2 |
|  | XP_028890274.1 | uncharacterized protein CJI97_003024 [[Candida] auris] | CauAto3 |
| *C. krusei* [5] | XP_029319197.1 | uncharacterized protein C5L36_0A03220 [Pichia kudriavzevii] | CkAto1 |
|  | XP_029320118.1 | uncharacterized protein C5L36_0A12180 [Pichia kudriavzevii] | CkAto2 |
|  | XP_029320117.1 | uncharacterized protein C5L36_0A12170 [Pichia kudriavzevii] | CkAto3 |
|  | XP_029320119.1 | uncharacterized protein C5L36_0A12190 [Pichia kudriavzevii] | CkAto4 |
|  | XP_029319710.1 | uncharacterized protein C5L36_0A08310 [Pichia kudriavzevii] | CkAto5 |
| *C. guilliermondii* [3] | EDK38382.1 | hypothetical protein PGUG_02480 [Meyerozyma guilliermondii ATCC 6260] | CguAto1 |
|  | EDK40033.2 | hypothetical protein PGUG_04131 [Meyerozyma guilliermondii ATCC 6260] | CguAto2 |
|  | EDK36918.2 | hypothetical protein PGUG_01016 [Meyerozyma guilliermondii ATCC 6260] | CguAto3 |
| *C. lusitaniae* [4] | XP_002619494.1 | hypothetical protein CLUG_00653 [Clavispora lusitaniae ATCC 42720] | ClAto1 |
|  | XP_002618592.1 | hypothetical protein CLUG_02051 [Clavispora lusitaniae ATCC 42720] | ClAto2 |
|  | XP_002618879.1 | hypothetical protein CLUG_00038 [Clavispora lusitaniae ATCC 42720] | ClAto3 |
|  | XP_002617751.1 | hypothetical protein CLUG_01210 [Clavispora lusitaniae ATCC 42720] | ClAto4 |
| *C. parapsilosis* [7] | CCE40409.1 | hypothetical protein CPAR2_104450 [Candida parapsilosis] | CpAto1 |
|  | CCE44056.1 | hypothetical protein CPAR2_502810 [Candida parapsilosis] | CpAto2 |
|  | CCE44055.1 | hypothetical protein CPAR2_502800 [Candida parapsilosis] | CpAto3 |
|  | CCE44009.1 | hypothetical protein CPAR2_502340 [Candida parapsilosis] | CpAto4 |
|  | CCE45313.1 | hypothetical protein CPAR2_703260 [Candida parapsilosis] | CpAto5 |
|  | CCE39768.1 | hypothetical protein CPAR2_601880 [Candida parapsilosis] | CpAto6 |
|  | CCE44054.1 | hypothetical protein CPAR2_502790 [Candida parapsilosis] | CpAto7 |
| *C. dubliniensis* [8] | XP_002418576.1 | acetate transporter, putative [Candida dubliniensis CD36] | CdAto1 |
|  | XP_002419032.1 | acetate transporter, putative [Candida dubliniensis CD36] | CdAto2 |
|  | XP_002419033.1 | ammonia export protein, putative [Candida dubliniensis CD36] | CdAto3 |
|  | XP_002419463.1 | acetate transporter, putative [Candida dubliniensis CD36] | CdAto4 |
|  | XP_002419461.1 | acetate transporter, putative [Candida dubliniensis CD36] | CdAto5 |
|  | XP_002421693.1 | acetate transporter, putative [Candida dubliniensis CD36] | CdAto6 |
|  | XP_002420929.1 | ammonia transport protein, putative [Candida dubliniensis CD36] | CdAto7 |
|  | XP_002418190.1 | acetate transporter, putative [Candida dubliniensis CD36] | CdAto8 |
| *C. tropicalis* [8] | [XP_002547488.1](https://www.ncbi.nlm.nih.gov/protein/XP_002547488.1?report=genbank&log$=prottop&blast_rank=1&RID=VKSBP26B014) | protein FUN34 [Candida tropicalis MYA-3404] | CtAto1 |
|  | [XP_002549734.1](https://www.ncbi.nlm.nih.gov/protein/XP_002549734.1?report=genbank&log$=prottop&blast_rank=2&RID=VKSBP26B014) | protein FUN34 [Candida tropicalis MYA-3404] | CtAto2 |
|  | [XP_002546727.1](https://www.ncbi.nlm.nih.gov/protein/XP_002546727.1?report=genbank&log$=prottop&blast_rank=3&RID=VKSBP26B014) | hypothetical protein CTRG_06205 [Candida tropicalis MYA-3404] | CtAto3 |
|  | [XP_002548540.1](https://www.ncbi.nlm.nih.gov/protein/XP_002548540.1?report=genbank&log$=prottop&blast_rank=4&RID=VKSBP26B014) | conserved hypothetical protein [Candida tropicalis MYA-3404] | CtAto4 |
|  | [XP_002546210.1](https://www.ncbi.nlm.nih.gov/protein/XP_002546210.1?report=genbank&log$=prottop&blast_rank=5&RID=VKSBP26B014) | conserved hypothetical protein [Candida tropicalis MYA-3404] | CtAto5 |
|  | [XP_002548538.1](https://www.ncbi.nlm.nih.gov/protein/XP_002548538.1?report=genbank&log$=prottop&blast_rank=6&RID=VKSBP26B014) | conserved hypothetical protein [Candida tropicalis MYA-3404] | CtAto6 |
|  | XP_002547090.1 | conserved hypothetical protein [Candida tropicalis MYA-3404] | CtAto7 |
|  | XP_002546489.1 | conserved hypothetical protein [Candida tropicalis MYA-3404] | CtAto8 |
| *C. kefyr* [5] | XP_022674350.1 | hypothetical protein KLMA_20009 [Kluyveromyces marxianus DMKU3-1042] | CkeAto1 |
|  | XP_022674438.1 | hypothetical protein KLMA_20099 [Kluyveromyces marxianus DMKU3-1042] | CkeAto2 |
|  | XP_022678050.1 | hypothetical protein KLMA_70455 [Kluyveromyces marxianus DMKU3-1042] | CkeAto3 |
|  | XP_022674905.1 | ammonia transport outward protein 3 [Kluyveromyces marxianus DMKU3-1042] | CkeAto4 |
|  | XP_022674904.1 | hypothetical protein KLMA_20586 [Kluyveromyces marxianus DMKU3-1042] | CkeAto5 |
| *C. haemulonis* [3] | XP_025340936.1 | hypothetical protein CXQ85_001773 [[Candida] haemulonis] | ChAto1 |
|  | XP_025341341.1 | hypothetical protein CXQ85_002189 [[Candida] haemulonis] | ChAto2 |
|  | XP_025341450.1 | hypothetical protein CXQ85_002302 [[Candida] haemulonis] | ChAto3 |
| *C. orthopsiliosis* [7] | XP_003867713.1 | Frp3 ammonium transporter [Candida orthopsilosis Co 90-125] | CoAto1 |
|  | XP_003867405.1 | hypothetical protein CORT_0B02520 [Candida orthopsilosis Co 90-125] | CoAto2 |
|  | XP_003867454.1 | Ato2 fungal-specific transmembrane protein [Candida orthopsilosis Co 90-125] | CoAto3 |
|  | XP_003871155.1 | Yhr032w protein [Candida orthopsilosis Co 90-125] | CoAto4 |
|  | XP_003867455.1 | Ato1 fungal-specific transmembrane protein [Candida orthopsilosis Co 90-125] | CoAto5 |
|  | XP_003870637.1 | Frp6 protein [Candida orthopsilosis Co 90-125] | CoAto6 |
|  | XP_003871144.1 | hypothetical protein CORT_0G03430 [Candida orthopsilosis Co 90-125] | CoAto7 |
